# Efficacy and safety of venlafaxine hydrochloride combined with tandospirone citrate for patients with vascular depression accompanied by somatic symptoms: An open‐labeled randomized control trial

**DOI:** 10.1111/cns.14650

**Published:** 2024-03-21

**Authors:** Hongbin Chen, Yongsen Lin, Zijun Zhao, Ting Lin, Qianwen Lin, Xinyan Chen, Weiwei Wu, Guiying Zeng, Shufang Wu, Nan Liu, Hui Chen, Ronghua Chen, Yingchun Xiao

**Affiliations:** ^1^ Department of Neurology Fujian Medical University Union Hospital Fuzhou China; ^2^ Department of Neurology First Hospital of Quanzhou Affiliated to Fujian Medical University Quanzhou China; ^3^ Gynecology Department, Fujian Maternity and Child Health Hospital Affiliated Hospital of Fujian Medical University Fuzhou China

**Keywords:** anxiety, somatic symptoms, tandospirone, vascular depression, venlafaxine

## Abstract

**Aims:**

To explore the pharmacological treatment of vascular depression (VaDep) and whether the blood levels of neurotransmitters can reflect the VaDep severity.

**Methods:**

VaDep patients with somatic symptoms were enrolled and randomly received venlafaxine + tandospirone (Combined Group) or venlafaxine (Monotherapy Group). The treatment efficacy was assessed by Hamilton Depression Scale (HAMD), Hamilton Anxiety Scale (HAMA), and Patient Health Questionnaire‐15 (PHQ‐15). The levels of blood monoamine neurotransmitters were measured by enzyme‐linked immunosorbent assay.

**Results:**

Both groups reported a progressive decrease in HAMD, HAMA, and PHQ‐15 scores to below the baseline after the respective treatment. Compared with the Monotherapy Group, the Combined Group reported a significant decrease in HAMD score at week 2 and markedly lower HAMA and PHQ‐15 scores at weeks 1, 2, 4, and 8. Both groups showed a decrease in the levels of blood monoamine neurotransmitters at weeks 4 and 8 when compared with the baseline. A strong positive association was evident between the plasma 5‐HT levels and the HAMD score.

**Conclusion:**

The combined therapy rapidly acts on VaDep comorbid with anxiety and somatic symptoms and significantly alleviates the anxiety and somatic symptoms. The plasma levels of 5‐HT may serve as potential objective candidates in evaluating VaDep severity and the efficacy of the undertaken treatment regimen.

## Graphical abstract

The changes in HAMD, HAMA, PHQ‐15 scores, and blood indexes in patients with vascular depression accompanied by somatic symptoms before and after treatment.

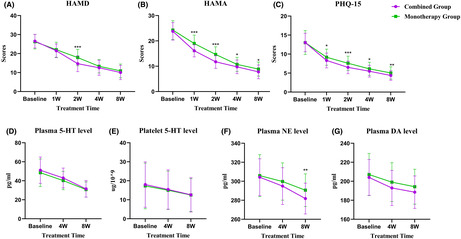

## 1 INTRODUCTION

Vascular depression (VaDep), a subtype of late‐life depression, is a geriatric depressive syndrome closely associated with cerebrovascular diseases or vascular risk factors.^1^, ^2^, ^3^ A previous survey showed that the VaDep incidence rate in the United States was about 3.4%.^4^

Compared with non‐vascular depression, VaDep features unique clinical characteristics, characteristic of more mental and physical symptoms, including extreme apathy, psychomotor retardation, impaired executive function, and chronic pain.^5^ In its acute stage, these symptoms are often accompanied by anxiety, which leaves such patients more susceptible to severe psychosomatic symptoms, worse social dysfunction, and higher suicidal tendency.^3^

To date, the specific mechanism underlying VaDep has largely remained undefined. Several hypotheses have been proposed to explain the pathogenesis of VaDep, including ischemia‐induced white matter lesions, inflammation, and hypoperfusion.^5^ Other studies suggest that depression and anxiety are mainly caused by the metabolic disorder of the neurotransmitters, including serotonin (5‐HT) and norepinephrine (NE), which has found substantial support in the findings regarding the action of antidepressants.^6^

Compared with the healthy population, patients with non‐vascular depression have a higher platelet 5‐HT level, which can be lowered by anti‐depression treatment.^7^ Therefore, platelets can serve as a peripheral index for changes in neural transmitters.^7^ Existent studies have reported that patients with depression have higher levels of plasma 5‐HT, NE, and dopamine (DA).^8^, ^9^, ^10^ However, it remains obscure whether the neurotransmitters in the plasma and platelets can reflect the severity of VaDep. So far, the severity of VaDep is generally evaluated with Hamilton Depression Scale (HAMD),^11^ which carries a strong bearing of subjectivity. Therefore, an exploration of objective indicators may hold great promise for the assessment of the clinical efficacy of VaDep treatments.

Patients with VaDep respond poorly to mono‐antidepressant treatments and are more likely to suffer from persistent depressive symptoms, which is termed as a treatment‐resistant depression.^5^ In contrast with the mono‐antidepressant treatment, combination therapies have demonstrated significant efficacy in tackling treatment‐resistant depression and can sustain adequate safety and medication adherence.^12^, ^13^, ^14^ A preliminary study reveals that for VaDep patients, a treatment with escitalopram plus tandospirone citrate can shorten the onset time of action on depression and ameliorate anxiety symptoms and cognitive dysfunction, though with no effective alleviation of their somatic symptoms.^15^ Therefore, it is of great significance to find a therapeutic candidate that can rapidly improve the somatic symptoms in VaDep patients.

Alternatively, as a selective serotonin and norepinephrine reuptake inhibitor (SNRI), venlafaxine has been found to be effective against depression and anxiety when compared with selective serotonin reuptake inhibitors (SSRIs).^16^ Venlafaxine can also alleviate somatic symptoms, such as chronic pain, in patients with depression.^17^ On the contrary, as a selective 5‐HT_1A_ receptor partial agonist and a synergist, tandospirone citrate also boasts significant anti‐anxiety effects^12^ and can shorten the onset time of action and enhance the effectiveness of antidepressants.^15^ Therefore, it is often used as an adjunct to the first‐line antidepressants. We, therefore, undertook an open‐labeled randomized control trial (RCT) and speculated that a regimen of tandospirone citrate plus venlafaxine might have sound efficacy against VaDep, quicken the onset of action of venlafaxine, and enhance the therapeutic effect of venlafaxine on anxiety and somatic symptoms in VaDep patients.

## 2 MATERIALS AND METHODS

### 2.1 Research participants

This was an open‐labeled RCT, enrolling patients who visited the neurology clinic or were admitted to the inpatient department of Fujian Medical University Union Hospital from October 2017 to December 2019. The inclusion criteria were as follows: (1) meeting the criteria of Diagnostic and Statistical Manual of Mental Disorders 4th edition (DSM‐IV)^18^ for a first episode or relapse of depression; (2) diagnosis conformed to the diagnostic standard for VaDep^1^; (3) a score of ≥17 in the 17‐item Hamilton Depression Rating Scale (HAMD‐17)^11^; (4) a score of ≥14 in the 14‐item Hamilton Anxiety Scale (HAMA‐14)^19^; (5) a score of ≥5 in Patient Health Questionnaire‐15 (PHQ‐15)^20^; and (6) a clear mind and capability of answering all neurological scales independently. The patients were excluded from the study due to any of the following exclusion criteria: (1) psychological diseases other than depression, anxiety, and somatic symptoms; (2) severe physical diseases, such as heart, liver, kidney, and respiratory diseases; (3) other nerve system diseases, such as Alzheimer's disease, Parkinson's disease, and intracranial tumor; (4) failure to undergo head magnetic resonance imaging scan; (5) allergy to venlafaxine or tandospirone; (6) a high suicidal tendency (scoring more than 30 points) in the Suicide Assessment Scale^21^; (7) proven alcohol or drug dependence (scoring 5 points or more) according to the Michigan Alcoholism Screening Test^22^; (8) participation in other clinical studies in the last 30 days; or (9) poor medication adherence (scoring less than 6 points) according to the Morisky Medication Adherence Scale‐8.^23^

This study was approved by the ethics committee of Fujian Medical University Union Hospital (Approval No.: 2013038) and registered with chictr.org.cn (Registration NO.: ChiCTR2100048442, title: Clinical study of venlafaxine hydrochloride combined with tandospirone citrate in the treatment of VaDep patients with somatic symptoms). The informed consent was obtained from all enrolled patients.

### 2.2 Sample size and grouping

This study was an exploratory trial, with no data reference, making an accurate estimation of the sample size difficult. Ultimately, a total of 198 participants were enrolled, of whom 136 were finally included in the study according to the inclusion and exclusion criteria. The participants were randomly divided into two groups: the Combined Group, receiving venlafaxine hydrochloride and tandospirone citrate; and the Monotherapy Group, receiving venlafaxine hydrochloride only. Participants were randomly allocated to each group using a 1:1 allocation algorithm generated by SPSS 25.0 software (SPSS Inc, IL, USA) and assigned by an independent investigator blinded to patient information. Randomization information for each eligible patient was sealed in opaque envelopes corresponding to the patient's registration number. The efficacy of the treatment schemes was assessed by two evaluators (one senior attending neurologist and one associate chief neurologist who were qualified for neuropsychological evaluation), who were trained in scale consistency and blinded to the grouping and medication of patients.

### 2.3 Drug therapy

Before the treatment, all included participants underwent a 1‐week drug washout (2 weeks for patients who took monoamine oxidase inhibitors and long half‐life antidepressants). The Combined Group was given a dose of 75 mg of venlafaxine hydrochloride in sustained‐release capsules (Pfizer Ireland Pharmaceuticals, USA) once a day. If no adverse events (AEs) occurred, the dosage was adjusted to 150 mg once a day at week 2. These patients also received, from the start of the therapy, 5 mg of tandospirone citrate (Sumitomo Pharmaceuticals Co., Ltd., Suzhou, China) three times a day, which was adjusted to 10 mg three times a day at week 2. The Monotherapy Group received 75 mg of venlafaxine hydrochloride (Pfizer Ireland Pharmaceuticals, USA) once a day. If no AEs occurred, the dosage was changed to 150 mg once a day from week 2. If any AEs developed during venlafaxine administration, the dose of venlafaxine was reduced by half and given once a day. Then, the patient was observed for 4 consecutive days. If the AE was alleviated, the dose was gradually increased to 150 mg once a day. The drug treatment spanned 8 weeks in both the combined cohort and the monotherapy cohort.

During the treatment, patients were prohibited from taking any triptans, other antipsychotics, catecholamines, glucocorticoids, monoamine oxidase inhibitors, and β‐receptor blockers. They were allowed to take antiplatelet drugs, anticoagulants, vitamin B, angiotensin‐converting enzyme inhibitors, angiotensin receptor blockers, and calcium channel antagonists.

### 2.4 Baseline demographic and clinical characteristics

The baseline characteristics recorded in this study included age, sex, education, course of disease, Fazekas score,^24^ scores of HAMD, HAMA, PHQ‐15, and the levels of plasma and platelet 5‐HT and plasma NE and DA.

### 2.5 Follow‐up visits, efficacy evaluation, and AEs

The follow‐up visits in this study lasted for 8 weeks. The scales of HAMD, HAMA, PHQ‐15, and Clinical Global Impression (CGI)^25^ were used to evaluate the treatment efficacy at weeks 1, 2, 4, and 8 after the administration. The effectiveness in alleviating depressive symptoms and somatization symptoms was designated as a respective decrease of at least 50% in the HAMD‐17 and PHQ‐15 total scores from baseline. For anxiety symptoms, a decrease of at least 40% in the HAMA‐14 total score from baseline was required.

The primary outcome was changes in PHQ‐15 score at week 8 after the treatment, in comparison with the baseline. The secondary outcome indices were the changes in the HAMD score, HAMA score, CGI scores, and blood monoamine neurotransmitter levels at weeks 1, 2, 4 and 8 after the treatment, as well as the reduction rates of HAMD, HAMA, and PHQ‐15 scores at weeks 1, 2, 4, and 8 after the treatment, in comparison with those of the baseline, respectively. The patients' blood pressure, blood routine indexes, liver function, renal function, electrolytes, and electrocardiogram were monitored during the treatment. The severity of AEs was evaluated with the Treatment Emergent Symptom Scale (TESS).^26^

### 2.6 Blood index collection and testing

#### 2.6.1 Blood sample collection

The morning fasting blood samples of all participants were obtained before the treatment and at weeks 4 and 8 after the treatment. Patients were required not to consume foods rich in tryptophan or tyrosine 72 h before blood collection, in that the intake of foods rich in such amino acids might affect the levels of plasma 5‐HT, NE, and DA.^27^, ^28^ Participants were also required to avoid food from 22:00 on the day before the blood collection and to relax and rest for 30 min from 08:00 to 10:00 a.m. on the day of the blood collection. Morning fasting venous blood was collected in two glass tubes containing ethylenediaminetetraacetic acid (3 mL into each tube) for subsequent platelet and plasma extraction.

#### 2.6.2 Platelet and plasma extraction

The platelets were extracted with the human peripheral‐blood platelet separation solution (Batch No.: PLA2014TBD; Tianjin Haoyang Biological Manufacture Co., Ltd., China). The centrifugal extraction of platelets and plasma was performed with low‐speed table‐top centrifuges (Model No.: TDL‐50B; Shanghai Anting Scientific Instrument Factory, China). The extracted platelets and plasma were placed in a −80°C refrigerator for a period of no more than 2 months. The levels of 5‐HT in the plasma and platelets were detected with an enzyme‐linked immunosorbent assay (ELISA) kit for calcitonin gene–related peptide (Cloud‐Clone Corp., Wuhan, China, No.: CEA876Hu 96T).^29^ The levels of NE and DA in the plasma were respectively measured with an ELISA kit for noradrenaline (Cloud‐Clone Corp., No.: CEA907Ge 96T) and one for dopamine (Cloud‐Clone Corp., No.: CEA851Ge 96T).^30^

### 2.7 Statistical analysis

All data were examined for normality and analyzed with SPSS 25.0 software (SPSS Inc., IL, USA). The missing data were filled by regression imputation. The primary and secondary outcomes were subjected to the intention‐to‐treat (ITT) analysis. Primary endpoint's variables were examined for normality by inspecting Q‐Q plots and frequency histograms and by the Kolmogorov–Smirnov test. All measurement data were expressed as mean ± standard deviation (*x* ± *s*). Between‐group and within‐group variables were analyzed by the repeated measures analysis of variance. All enumeration data were expressed as ratios (percentages) and compared by the chi‐squared test or Fisher's extract test. The correlations between HAMD, HAMA, PHQ‐15, and blood indexes were respectively analyzed by partial Spearman correlation analysis after adjustment for age, sex, course, education years, group, and levels of other monoamine neurotransmitters. Bilateral *p* < 0.05 indicated a statistical significance.

## 3 RESULTS

### 3.1 Research participants

**FIGURE 1:**
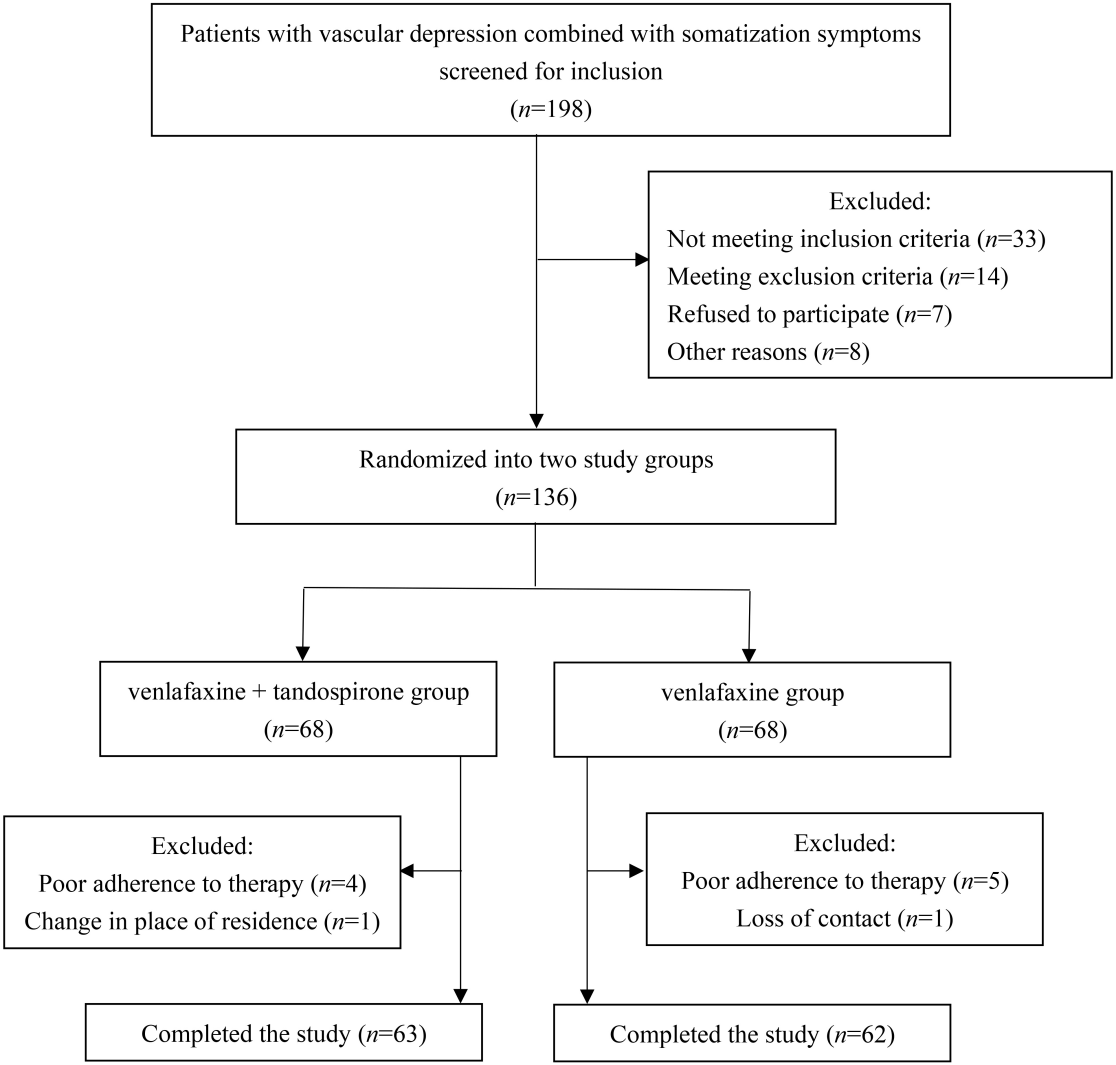
Flowchart of patient inclusion.

Follow‐up visits of patients were recorded according to the flowchart shown in Figure 1. Of the total 198 participants in the study, 62 were excluded and the remaining 136 were randomly divided into the Combined Group and the Monotherapy Group, each group comprising 68 participants. During the 8‐week follow‐up visit, 5 participants in the Combined Group and 6 participants in the Monotherapy Group dropped out of the study.

### 3.2 Baseline characteristics of participants

The baseline characteristics of the participants are shown in Table 1. No significant differences in baseline characteristics were found between the two groups (*p* > 0.05).

**TABLE 1:** Baseline characteristics of participants.

Baseline characteristic	Combined group (*N* = 68)	Monotherapy group (*N* = 68)
Age (year)	72.13 ± 5.45	71.82 ± 5.05
Sex (male/female)	32/36	35/33
Education (year)	7.72 ± 2.51	7.94 ± 2.23
Course of disease (year)	3.93 ± 2.52	3.88 ± 2.34
Fazekas score (point)	2.01 ± 0.82	2.04 ± 0.76
HAMD score (point)	26.56 ± 3.60	26.24 ± 3.84
HAMA score (point)	23.78 ± 3.46	24.28 ± 3.72
PHQ‐15 score (point)	13.04 ± 2.42	13.03 ± 3.16
Plasma 5‐HT level (pg/mL)	51.13 ± 13.89	48.48 ± 14.50
Platelet 5‐HT level (μg/10^9^)	18.00 ± 11.94	17.29 ± 12.00
Plasma NE level (pg/mL)	304.28 ± 19.39	305.96 ± 22.13
Plasma DA level (pg/mL)	204.02 ± 18.94	207.13 ± 22.23

*Note*: Data are shown as mean ± standard deviation or *n*.

Abbreviations: 5‐HT, 5‐hydroxytryptamine; DA, dopamine; HAMA, Hamilton Anxiety Scale; HAMD, Hamilton Depression Scale; NE, norepinephrine; PHQ‐15, Patient Health Questionnaire‐15.

### 3.3 Comparison of treatment efficacy against depression, anxiety, and somatic symptoms between the two groups

For both the Combined Group and the Monotherapy Group, repeated measures analysis of variance was conducted for each score at each time point. The results reported a significant main effect of time, but not that of grouping, for HAMD‐17 scores; a significant main effect of both time and grouping for HAMA‐14 and PHQ‐15 scores; and a significant time × group interaction for HAMD‐17, HAMA‐14, and PHQ‐15 scores, respectively (Table 2).

**TABLE 2:** The results of repeated measures analysis of variance between two groups and Cohen's *d* with 95% CI of two groups at each time point.

Outcome	Time		Group		Time × group interaction		Cohen's *d* effect size (95% CI)			
	*F*	*p*	*F*	*p*	*F*	*p*	1 week	2 weeks	4 weeks	8 weeks
HAMD	1480.096	**<0.001**	3.065	0.082	15.521	**<0.001**	−0.152 (−0.489, 0.185)	**−0.773 (−1.120, −0.423)**	−0.200 (−0.537, 0.137)	−0.232 (−0.569, 0.106)
HAMA	929.234	**<0.001**	2.037	**<0.001**	7.515	**<0.001**	**−0.980 (−1.335, −0.623)**	**−1.025 (−1.382, −0.666)**	**−0.353 (−0.691, −0.014)**	**−0.398 (−0.737, −0.058)**
PHQ‐15	786.964	**<0.001**	5.627	**0.019**	3.134	**0.015**	**−0.419 (−0.758, −0.079)**	**−0.615 (−0.958, −0.270)**	**−0.386 (−0.724, −0.046)**	**−0.466 (−0.806, −0.124)**
Platelet 5‐HT level	145.728	**<0.001**	0.055	0.814	0.503	0.605	n/a	n/a	0.272 (−0.066, 0.609)	0.093 (−0.244, 0.429)
Plasma 5‐HT level	137.508	**<0.001**	2.151	0.145	0.477	0.621	n/a	n/a	0.041 (−0.296, 0.377)	0.013 (−0.324, 0.349)
Plasma NE level	207.622	**<0.001**	2.732	0.101	7.404	**0.001**	n/a	n/a	−0.247 (−0.584, 0.091)	**−0.529 (−0.870, −0.186)**
Plasma DA level	189.959	**<0.001**	2.447	0.120	2.506	0.083	n/a	n/a	−0.316 (−0.654, 0.023)	−0.324 (−0.662, 0.014)

*Note*: Bold values indicate statistically significant results.

Abbreviations: 5‐HT, 5‐hydroxytryptamine; DA, dopamine; HAMA, Hamilton Anxiety Scale; HAMD, Hamilton Depression Scale; NE, norepinephrine; PHQ‐15, Patient Health Questionnaire‐15.

**FIGURE 2:**
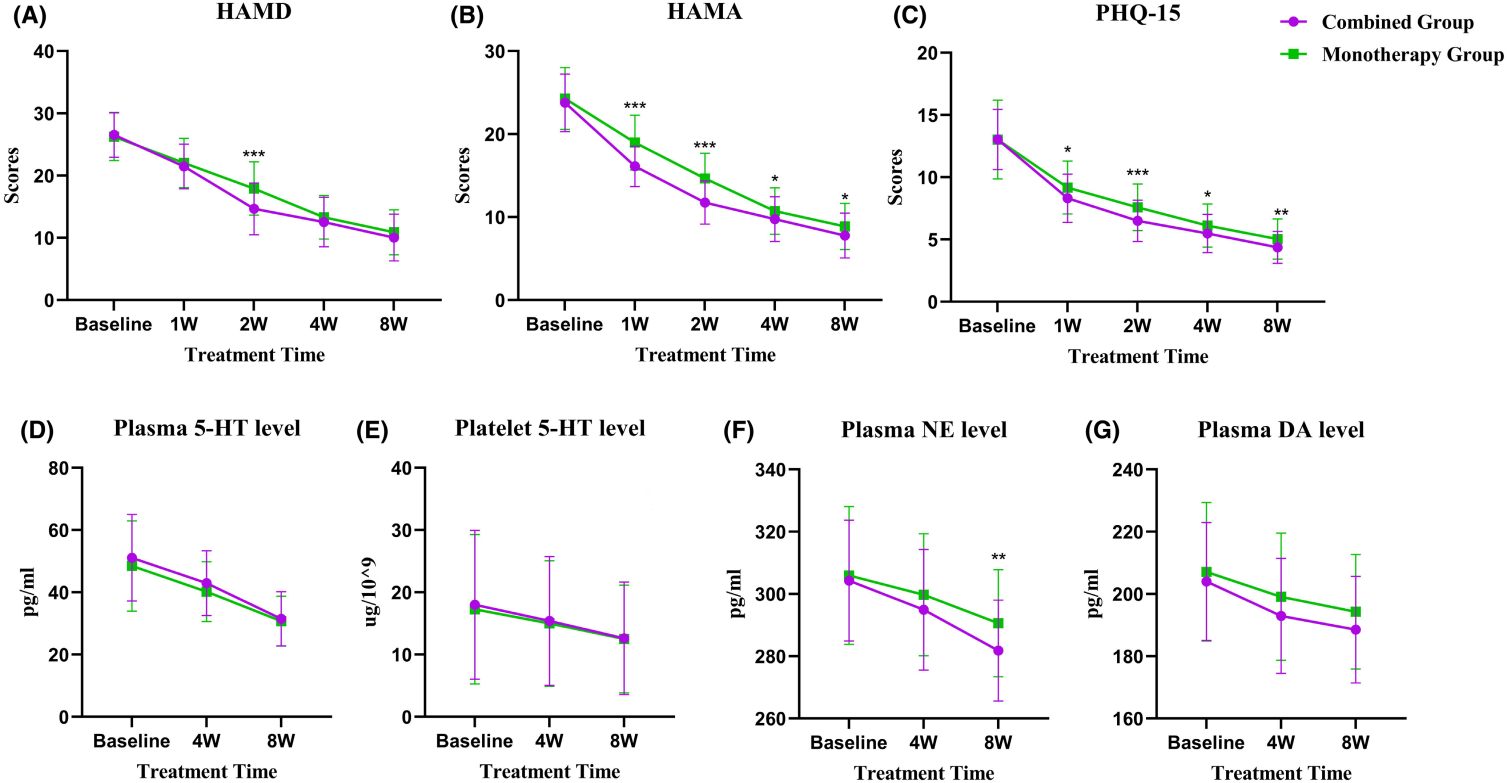
(A) Comparison of HAMD scores between the two groups. (B) Comparison of HAMA scores between the two groups. (C) Comparison of PHQ‐15 scores between the two groups. (D) Comparison of plasma 5‐HT levels between the two groups. (E) Comparison of platelet 5‐HT levels between the two groups. (F) Comparison of plasma NE levels between the two groups. (G) Comparison of plasma DA levels between the two groups. 5‐HT, serotonin; DA, dopamine; HAMA, Hamilton Anxiety Scale; HAMD, Hamilton Depression Rating Scale; NE, norepinephrine; PHQ‐15, Patient Health Questionnaire‐15. *p < 0.05, **p < 0.01, and ***p < 0.001.

After the treatment, Cohen's *d* effect size for comparing HAMD‐17, HAMA‐14, and PHQ‐15 scores at each time point between the Combined Group and the Monotherapy Group was depicted in Table 2. Compared with those before the treatment, HAMD‐17, HAMA‐14, and PHQ‐15 scores decreased in both groups after the treatment; compared with the Monotherapy Group, the Combined Group reported significantly lower HAMD‐17 scores at week 2 and markedly lower HAMA‐14 and PHQ‐15 scores at weeks 1, 2, 4, and 8 (Figure 2 and Table S1).

The reduction rates of HAMD, HAMA, and PHQ‐15 scores at each time point after the treatment are summarized in Table S2. The results showed that the reduction rate of the HAMD scores exceeded 50% after 4 weeks of treatment in the Combined Group and after 8 weeks of treatment in the Monotherapy Group; and a similar reduction rate of the HAMA and PHQ‐15 scores appeared after 2 weeks of treatment in the Combined Group and after 4 weeks of treatment in the Monotherapy Group.

### 3.4 The changes in the levels of platelet 5‐HT, plasma 5‐HT, plasma NE, and plasma DA before and after the treatment

As shown in Table 2, a significant main effect of time was found for the levels of platelet 5‐HT, plasma 5‐HT, plasma NE, and plasma DA, but no main effect of grouping was evident. A significant time × group interaction was reported for plasma NE. The Cohen's *d* effect size was performed to compare the levels of platelet 5‐HT, plasma 5‐HT, plasma NE, and plasma DA between the Combined Group and the Monotherapy Group at each time point after treatment (Table 2). Additionally, both groups showed a decrease in the levels of platelet 5‐HT, plasma 5‐HT, plasma NE, and plasma DA after the treatment when in comparison with those before the treatment. At weeks 4 and 8, the two groups reported no significant differences in the levels of platelet 5‐HT, plasma 5‐HT, and DA. However, compared with the Monotherapy Group, the Combined Group exhibited a significantly lower level of plasma NE at week 8 (Figure 2 and Table S1).

### 3.5 Analysis of correlations between blood indexes and HAMD, HAMA, and PHQ‐15 scores

**FIGURE 3:**
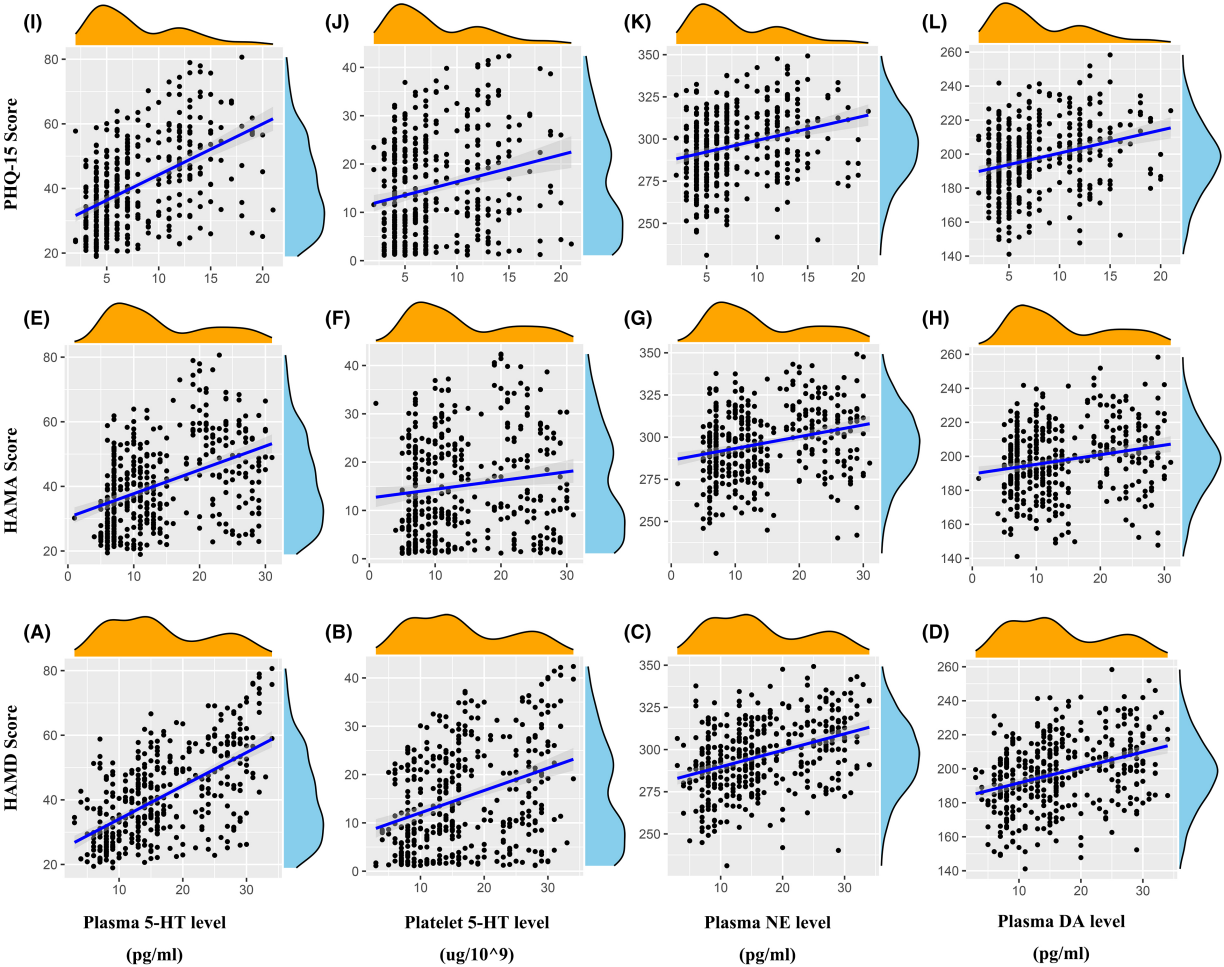
(A) Correlation between plasma 5‐HT level and HAMD score. (B) Correlation between platelet 5‐HT level and HAMD score. (C) Correlation between plasma NE level and HAMD score. (D) Correlation between plasma DA level and HAMD score. (E) Correlation between plasma 5‐HT level and HAMA score. (F) Correlations between platelet 5‐HT level and HAMA score. (G) Correlations between plasma NE level and HAMA score. (H) Correlation between plasma DA level and HAMA score. (I) Correlation between plasma 5‐HT level and PHQ‐15 score. (J) Correlations between platelet 5‐HT level and PHQ‐15 score. (K) Correlation of plasma NE level and PHQ‐15 score. (L) Correlation of plasma DA level and PHQ‐15 score. Plasma 5‐HT level was positively correlated with HAMD score (*r* = 0.615, *p* < 0.001); platelet 5‐HT, plasma NE and DA levels were weakly correlated with HAMD score; and plasma 5‐HT level weakly correlated with HAMA and PHQ‐15 scores. 5‐HT, serotonin; DA, dopamine; HAMA, Hamilton Anxiety Scale; HAMD, Hamilton Depression Rating Scale; NE, norepinephrine; PHQ‐15, Patient Health Questionnaire‐15.

The correlations between blood indexes and HAMD, HAMA, and PHQ‐15 scores at baseline, weeks 4 and 8 after the treatment in the two groups are shown in Figure 3 and Table 3. The HAMD scores were significantly positively correlated with the levels of plasma 5‐HT and mildly correlated with the levels of platelet 5‐HT, plasma NE, and plasma DA. The HAMA scores were moderately correlated with the level of plasma 5‐HT and slightly with the levels of platelet 5‐HT, plasma NE, and plasma DA. The PHQ‐15 scores were moderately correlated with the levels of plasma 5‐HT and slightly with the levels of platelet 5‐HT, plasma NE, and plasma DA.

**TABLE 3:** Analysis of the correlation between blood indexes and HAMD, HAMA, and PHQ‐15.

Item (*r*‐value)	Plasma 5‐HT level	Platelet 5‐HT level	Plasma NE level	Plasma DA level
HAMD score	0.581***	0.338***	0.371***	0.360***
HAMA score	0.426***	0.148**	0.260***	0.206***
PHQ‐15 score	0.499***	0.219***	0.300***	0.290***

Abbreviations: 5‐HT, serotonin; DA, dopamine; HAMA, Hamilton Anxiety Scale; HAMD, Hamilton Depression Rating Scale; NE, norepinephrine; PHQ‐15, Patient Health Questionnaire‐15.

***p < 0.001, **p < 0.01.

### 3.6 Adverse drug events

The incidence of AEs in the two groups at each time point after the treatment is shown in Table S3. The total incidence of AEs was 35.29% in the Combined Group and 32.35% in the Monotherapy Group, without a significant difference between the two groups. AEs usually occur at the beginning of medication administration and with the dose increase. Patients developed mild symptoms (mostly scoring 1–2 points in TESS), which alleviated or disappeared after dose adjustment. AEs in the Combined Group included gastrointestinal discomfort (20 participants), dizziness (3 participants), and drowsiness (1 participant), while those of the Monotherapy Group included gastrointestinal discomfort (18 participants), dizziness (2 participants), and weakness (2 participants). None of the patients in both groups had obvious abnormalities in blood pressure, blood routine indexes, liver function, renal function, electrolyte, and ECG.

### 3.7 CGI scores

The CGI scores in the two groups at each time point after the treatment are shown in Table S4 and Figure S1. Compared with those before the treatment, the CGI‐S scores decreased in both groups at weeks 1, 2, 4, and 8 after the treatment (*p* < 0.001). CGI‐S score was significantly lower in the Combined Group than in the Monotherapy Group at weeks 1, 2, and 4 after the treatment (*p* < 0.05), with no significant difference between the two groups at week 8 after the treatment. CGI‐G score was significantly lower in the Combined Group than in the Monotherapy Group at weeks 1 and 2 after the treatment (*p* < 0.001). CGI‐E score was statistically higher in the Combined Group than in the Monotherapy Group at weeks 1 and 2 after the treatment (*p* < 0.05). CGI‐G and CGI‐E scores were not significantly different between the two groups at weeks 4 and 8 after the treatment.

## 4 DISCUSSION

In this open‐labeled RCT, compared with the venlafaxine monotherapy, the combined regimen of venlafaxine + tandospirone produced a significantly quicker action on VaDep and the accompanying anxiety and somatic symptoms, demonstrating a better efficacy against the accompanying anxiety and somatic symptoms, as indicated by the scores of HAMD, HAMA, and PHQ‐15 after the 8‐week follow‐up visit. In both groups, the patients tolerated both drug therapies well. The levels of plasma and platelet 5‐HT and plasma NE and DA in both groups declined after the anti‐depression treatment. Moreover, the HAMD scores were significantly positively correlated with the level of plasma 5‐HT. These findings may highlight a potential promising objective indicator for evaluating the severity of depression.

According to previous studies, monotherapy acts slowly on VaDep.^5^ The possible explanation may be attributed to the presence of 5‐HT_1A_ and α2 autoreceptors on the presynaptic membrane, which produce negative feedback and negative regulatory effects on the release of neurotransmitters such as 5‐HT and NE.^31^, ^32^ It may take several weeks for such autoreceptors in the presynaptic membrane to be downregulated and desensitized after an anti‐depression treatment, prolonging the action onset time of anti‐depression therapy.^31^, ^32^

Consistent with the previous findings,^33^ in our study, we found that the combined use of tandospirone and venlafaxine rapidly ameliorated VaDep and anxiety symptoms, along with proper safety and tolerance. We speculate that the mechanism may lie in the highly selective action of tandospirone on postsynaptic 5‐HT_1A_ receptors and presynaptic 5‐HT_1A_ autoreceptors. On the one hand, 5‐HT_1A_ receptors acting on the postsynaptic membrane can play an antidepressant effect similar to 5‐HT, and on the other hand, 5‐HT_1A_ autoreceptors acting on the presynaptic membrane can accelerate their desensitization, reduce the negative feedback effect of 5‐HT on the presynaptic 5‐HT_1A_ autoreceptors, and rapidly increase the concentration of 5‐HT in the synaptic cleft, thereby accelerating the onset of action of SSRIs or SNRIs. In the meantime, tandospirone can also act on 5‐HT_1A_ autoreceptors on neuronal somatodendrites, reduce neuronal nerve impulses and 5‐HT release from neurons extracellularly, and increase 5‐HT transmission to the presynaptic membrane, therefore increasing presynaptic 5‐HT transmitter levels.^34^

In our study, we found that the combined use of tandospirone and venlafaxine rapidly ameliorated somatic symptoms, which might be explained by the fact that the two drugs alleviate the somatic symptoms through different mechanisms. Venlafaxine has been shown to improve depression‐associated pain symptoms, while tandospirone has been reported to lessen anxiety‐associated gastrointestinal discomfort.^35^, ^36^ We speculate that tandospirone enhances the effectiveness of venlafaxine by improving somatic symptoms and accelerating the action of venlafaxine on somatic symptoms in VaDep patients.

Previous studies have documented declined platelet 5‐HT level in patients with non‐vascular depression after anti‐depression treatment.^7^ In this study, the platelet 5‐HT level in VaDep patients also dropped after the anti‐depression treatment. This reduction in platelet 5‐HT may arise from the inhibition of the platelet membrane 5‐HT transporter (SERT) and has been observed during treatment with SSRIs, SNRIs, and most tricyclic antidepressants.^7^ Venlafaxine acts on SERTs on both the presynaptic membrane of serotonergic neurons and the platelet surface to inhibit the uptake of 5‐HT by platelets, thus reducing the platelet 5‐HT level. However, our study found that although platelet 5‐HT level decreased with the antidepressant treatment, the correlation between platelet 5‐HT level and the severity of VaDep was quite weak (*r* = 0.338, *p* < 0.001), which is not consistent with our previous speculation. The inconsistency may be related to the differences in disease characteristics between VaDep patients and non‐vascular depression patients, but the specific mechanism needs to be further explored.

Accumulative evidence suggests that a decrease in plasma 5‐HT, as done by SSRIs, is associated with ameliorated depression, and that a high baseline plasma 5‐HT level is associated with better SSRI responses.^8^, ^37^ Our study found that plasma 5‐HT level significantly decreased from the baseline after antidepressant treatment in VaDep patients. In the analysis of the correlation between plasma 5‐HT level and HAMD scores, we found a significant positive correlation between plasma 5‐HT and HAMD scores (*r* = 0.581, *p* < 0.001). This suggests that plasma 5‐HT level may reflect the severity of depression in VaDep patients to some extent and may be used as a potential objective indicator of the severity of VaDep. However, studies also report no significant association between plasma 5‐HT level and depression severity.^38^ The disparity may lie in the fact that different races and depression rating scales are used in these studies, as different scales have distinct validity and reliability in evaluating depression.^39^ Therefore, further studies are needed to reveal the specific mechanisms underlying the association between high plasma 5‐HT levels and depression outcomes.

Our study found that plasma NE and DA levels were correlated with depression severity in VaDep patients to some extent (*r* = 0.371, *p* < 0.001; *r* = 0.360, *p* < 0.001, respectively), with plasma NE levels significantly lower in the Combined Group than in the Monotherapy Group after the treatment. Recent studies have found that plasma norepinephrine and dopamine levels are significantly increased in patients with major depression.^10^ Another study documents a marked increase in plasma and urinary norepinephrine in patients with major affective disorders, such as depression, bipolar depression, and unipolar depression.^9^ Still another reports that plasma NE levels may increase in patients with generalized anxiety disorder when compared with normal people.^40^ However, we found that despite the positive correlation between plasma NE and DA levels and HAMD scores, no statistical significance was evident. Therefore, further investigations are awaited to explore whether plasma NE and DA levels can be used as indicators of the disease severity in VaDep patients.

In addition, we also analyzed the correlation between blood index and HAMA and PHQ‐15 scores. The results showed that plasma 5‐HT was moderately correlated with HAMA and PHQ‐15 scores, and that platelet 5‐HT, plasma NE and DA levels were weakly correlated with HAMA and PHQ‐15 scores. These results suggest that plasma 5‐HT can serve as a relatively specific indicator of depression severity in VaDep patients and as a potential objective candidate to evaluate VaDep severity and the efficacy of the treatments for the depression population.

Some limitations remain in this study: (1) It was a single‐center study with a small sample size. Hence, studies of a larger sample size are needed to explore the overall status of patients with VaDep. (2) This study only lasted for 8 weeks. So, it remains unraveled whether the venlafaxine + tandospirone therapy would have long‐term effectiveness against VaDep and whether further changes in the blood indexes could be found. (3) Patients diagnosed with VaDep needed treatment for ethical reasons. Therefore, placebo control group was not designed in this study.

In summary, this study evidences that a combined therapy of venlafaxine plus tandospirone can rapidly act on VaDep comorbid with anxiety and somatic symptoms and produce strong efficacy against anxiety, somatic symptoms, and subjective cognitive dysfunction. The level of plasma 5‐HT may serve as a potential objective candidate in assessing VaDep severity and the effectiveness of the treatment. More studies are required to define the long‐term benefits of the combined therapy and the related mechanism.

## AUTHOR CONTRIBUTIONS

Hongbin Chen and Yongsen Lin were responsible for study conception and coordination, experiment design and performance, data collection and analysis, and manuscript drafting; Zijun Zhao, Ting Lin, Qianwen Lin, Xinyan Chen, Weiwei Wu, Guiying Zeng, Shufang Wu, and Nan Liu carried out data collection and analysis. Yingchun Xiao, Ronghua Chen, and Hui Chen carried out data analysis and revised the manuscript. All authors reviewed the results and approved the final version of the manuscript.

## FUNDING INFORMATION

This study was supported by Excellent Young Scholars Cultivation Project of Fujian Medical University Union Hospital (No. 2022XH026), Joint Funds for the Innovation of Science and Technology, Fujian province (No. 2019Y9097, No. 2020Y9082, No. 2021Y9060), the Natural Science Foundation of Fujian Province (No. 2023J01648) and the Startup Fund for scientific research, Fujian Medical University (No. 2021QH1238, No. 2020QH1201).

## CONFLICT OF INTEREST STATEMENT

The authors confirm no conflict of interest.

## Supporting information


Data S1

## Data Availability

The data that support the findings of this study are available from the corresponding author. The data are not publicly available due to privacy or ethical restrictions.
