# Supplementary material for: Efficacy and safety of venlafaxine hydrochloride combined with tandospirone citrate for patients with vascular depression accompanied by somatic symptoms: An open‐labeled randomized control trial
Source: CNS Neurosci Ther. 2024 Mar 21;30(3):e14650. doi: 10.1111/cns.14650 (PMC10957720; doi:10.1111/cns.14650)
Supplement: Supplementary file 1 — Data S1 [file CNS-30-e14650-s001.docx]

**Results**

**Supplemental Table 1. The changes in scores and blood indexes between the two groups before and after the treatment**

|  | **Item** | **Combined Group  (N=68)** | **Monotherapy Group  (N=68)** | ***F*** | ***p*** |
| --- | --- | --- | --- | --- | --- |
| HAMD Scores | baseline | 26.56±3.60 | 26.24±3.84 | 0.257 | 0.613 |
|  | 1W | 21.47±3.58 *^a^* | 22.04±3.95 *^a^* | 0.789 | 0.376 |
|  | 2W | 14.68±4.15 *^ab^* | 17.93±4.27 *^ab^* | 20.309 | **＜0.001** |
|  | 4W | 12.56±3.97 *^abc^* | 13.31±3.51 *^abc^* | 1.364 | 0.245 |
|  | 8W | 10.06±3.74 *^abcd^* | 10.91±3.62 *^abcd^* | 1.828 | 0.179 |
| HAMA Scores | baseline | 23.78±3.46 | 24.28±3.72 | 0.659 | 0.418 |
|  | 1W | 16.13±2.44 *^a^* | 18.99±3.31 *^a^* | 32.678 | **＜0.001** |
|  | 2W | 11.76±2.60 *^ab^* | 14.66±3.04 *^ab^* | 35.755 | **＜0.001** |
|  | 4W | 9.76±2.70 *^abc^* | 10.74±2.80 *^abc^* | 4.235 | **0.042** |
|  | 8W | 7.79±2.69 *^abcd^* | 8.88±2.78 *^abcd^* | 5.384 | **0.022** |
| PHQ-15 Scores | baseline | 13.04±2.42 | 13.03±3.16 | 0.001 | 0.976 |
|  | 1W | 8.32±1.94 *^a^* | 9.18±2.12 *^a^* | 5.975 | **0.016** |
|  | 2W | 6.50±1.66 *^ab^* | 7.59±1.87 *^ab^* | 12.863 | **＜0.001** |
|  | 4W | 5.49±1.54 *^abc^* | 6.12±1.73 *^abc^* | 5.060 | **0.026** |
|  | 8W | 4.37±1.28 *^abcd^* | 5.04±1.61 *^abcd^* | 7.376 | **0.007** |
| Platelet 5-HT level(ug/10^9) | baseline | 18.00±11.94 | 17.29±12.00 | 0.120 | 0.730 |
|  | 4W | 15.41±10.35 *^c^* | 15.00±10.09 *^c^* | 0.056 | 0.813 |
|  | 8W | 12.61±9.03 *^cd^* | 12.50±8.66 *^cd^* | 0.005 | 0.941 |
| Plasma 5-HT level(pg/ml) | baseline | 51.13±13.89 | 48.48±14.50 | 1.177 | 0.280 |
|  | 4W | 42.95±10.40 *^c^* | 40.23±9.57 *^c^* | 2.512 | 0.115 |
|  | 8W | 31.53±8.69 *^cd^* | 30.76±7.99 *^cd^* | 0.291 | 0.590 |
| Plasma NE level(pg/ml) | baseline | 304.28±19.39 | 305.96±22.13 | 0.220 | 0.640 |
|  | 4W | 294.95±19.38 *^c^* | 299.76±19.59 *^c^* | 2.075 | 0.152 |
|  | 8W | 281.81±16.19 *^cd^* | 290.64±17.20 *^cd^* | 9.503 | **0.002** |
| Plasma DA level(pg/ml) | baseline | 204.02±18.94 | 207.13±22.23 | 0.771 | 0.381 |
|  | 4W | 192.95±18.48 *^c^* | 199.11±20.43 *^c^* | 3.400 | 0.067 |
|  | 8W | 188.54±17.10 *^cd^* | 194.30±18.40 *^cd^* | 3.580 | 0.061 |

HAMD**,** Hamilton Depression Rating Scale**;** HAMA**,** Hamilton Anxiety Scale**;** PHQ-15**,** Patient Health Questionnaire-15**;** 5-HT, 5-hydroxytryptamine**;** NE**,** norepinephrine**;** DA**,** dopamine**;** *a,* *p* < 0.05 as compared with the baseline; *b,* *p* < 0.05 as compared with that at week 1; *c,* *p* < 0.05 as compared with that at week 2; and *d*, *p* < 0.05 as compared with that at week 4.

**Supplemental Table 2. The reduction rate of HAMD, HAMA and PHQ-15 scores at each time point after the treatment**

|  | **Item** | **Combined Group  (%)** | **Monotherapy Group  (%)** | ***p*** |
| --- | --- | --- | --- | --- |
| HAMD Scores | 1W | 19.60±7.54 | 16.13±8.26 | **0.012** |
|  | 2W | 45.25±12.04 | 32.20±10.70 | **＜0.001** |
|  | 4W | 53.35±12.11 | 49.75±9.35 | 0.054 |
|  | 8W | 62.42±12.31 | 58.74±10.92 | 0.067 |
| HAMA Scores | 1W | 31.70±8.03 | 21.67±7.39 | **＜0.001** |
|  | 2W | 50.17±10.21 | 39.17±10.89 | **＜0.001** |
|  | 4W | 58.29±12.09 | 54.74±12.93 | 0.101 |
|  | 8W | 66.44±12.38 | 62.25±13.67 | 0.063 |
| PHQ-15 Scores | 1W | 36.44±8.78 | 28.87±9.72 | **＜0.001** |
|  | 2W | 50.12±9.67 | 41.18±9.78 | **＜0.001** |
|  | 4W | 57.39±11.48 | 52.44±10.14 | **0.009** |
|  | 8W | 65.74±11.67 | 59.84±13.54 | **0.006** |

HAMD**,** Hamilton Depression Rating Scale**;** HAMA**,** Hamilton Anxiety Scale**;** PHQ-15**,** Patient Health Questionnaire-15**.**

**Supplemental Table 3. Adverse drug events**

| **Time** | **Combined Group (*N* = 68)** | **Monotherapy Group (*N* = 68)** | ***c*² value** | ***p* value** |
| --- | --- | --- | --- | --- |
| 0–7 days | 19 (27.94%) | 18 (26.47%) | – | – |
| 8–14 days | 4 (5.88%) | 4 (5.88%) | – | – |
| 15–28 days | 1 (1.47%) | 0 (0%) | – | – |
| 29–56 days | 0 (0%) | 0 (0%) | – | – |
| 8 weeks | 24 (35.29%) | 22 (32.35%) | 0.131 | 0.717 |

Data are presented as n (%).


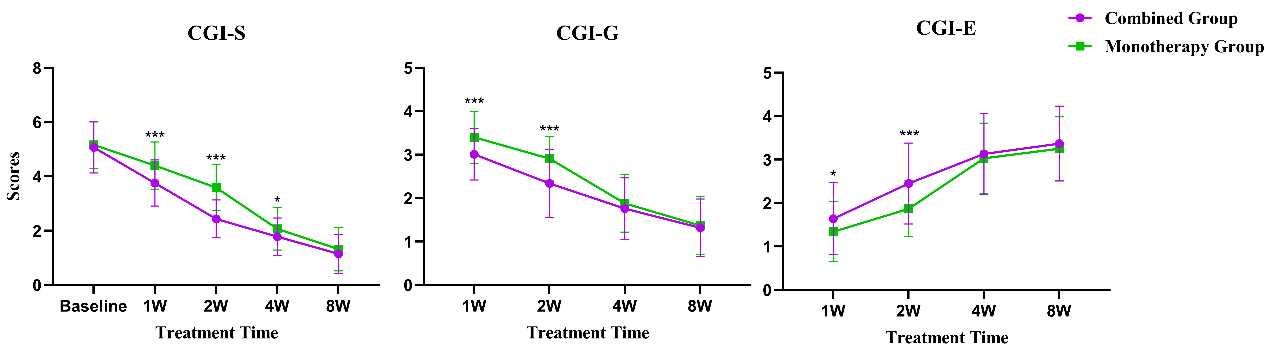


**Supplemental Figure 1. The comparison of CGI scores between the two groups.** ^*^*p* < 0.05, ^**^*p* < 0.01, and ^***^*p*< 0.001.

**Supplemental Table 4. CGI scores after the treatment**

| **Item** | **Time point** | **Combined Group (*N* = 68)** | **Monotherapy Group  (*N* = 68)** | **^†^*p* value** |
| --- | --- | --- | --- | --- |
| **CGI-S*^a^*** | Before treatment | 5.07±0.94 | 5.16±0.87 | 0.571 |
|  | 1 week after treatment | 3.76±0.85^#^ | 4.40±0.87^#^ | **＜0.001^***^** |
|  | 2 weeks after treatment | 2.44±0.70^#^ | 3.59±0.85^#^ | **＜0.001^***^** |
|  | 4 weeks after treatment | 1.78±0.69^#^ | 2.07±0.78^#^ | **0.021^*^** |
|  | 8 weeks after treatment | 1.15±0.72^#^ | 1.32±0.80^#^ | 0.178 |
| **CGI-G*^b^*** | 1 week after treatment | 3.01±0.59 | 3.40±0.60 | **＜0.001^***^** |
|  | 2 weeks after treatment | 2.34±0.78 | 2.91±0.51 | **＜0.001^***^** |
|  | 4 weeks after treatment | 1.76±0.71 | 1.88±0.66 | 0.320 |
|  | 8 weeks after treatment | 1.32±0.66 | 1.37±0.67 | 0.698 |
| **CGI-E*^c^*** | 1 week after treatment | 1.64±0.83 | 1.34±0.69 | **0.022^*^** |
|  | 2 weeks after treatment | 2.45±0.93 | 1.87±0.64 | **＜0.001^***^** |
|  | 4 weeks after treatment | 3.13±0.93 | 3.03±0.81 | 0.523 |
|  | 8 weeks after treatment | 3.37±0.86 | 3.25±0.74 | 0.395 |

*a*, Clinical Global Impression-Severity; *b*, Clinical Global Impression-General; *c*, Clinical Global Impression-Efficacy.

^#^ *p*< 0.001 as compared with that before the treatment.

^†^*P* value indicates the statistical difference between the venlafaxine + tandospirone group and the venlafaxine group at each time point.

^*^ *p* < 0.05, ^**^ *p* < 0.01, ^***^ *p* < 0.001.
